# Microscopic model for radiation-induced magnetoresistance oscillations excited by circularly polarized radiation

**DOI:** 10.1038/s41598-019-46057-3

**Published:** 2019-07-03

**Authors:** Jesús Iñarrea

**Affiliations:** 1 Escuela Politécnica Superior, Universidad Carlos III, 28911 Leganes, Madrid, Spain; 2 Unidad Asociada al Instituto de Ciencia de Materiales, CSIC Cantoblanco, Madrid, 28049 Spain

**Keywords:** Two-dimensional materials, Quantum dots, Two-dimensional materials, Quantum dots

## Abstract

We develop a microscopic model to explain the striking result of immunity to the sense of circularly polarized radiation of the photo-excited resistance oscillations in high-mobility 2D electron systems. Our model is based on the radiation-driven electron orbit model, previously developed to explain the photo-induced resistance oscillations and zero resistance states in these systems. According to it, the guiding center of the Landau states when irradiated by circularly polarized radiation performs a circular path driven by radiation. In principle, in an infinite sample, this path is different according to the the sense of circular polarization (left or right). However, the limited size of the sample with the essential role of the edges and the concurrent presence of the Hall electric field tend to quench the displacement of the driven guiding center making nearly equal both trajectories. In the end and in the presence of scattering, the longitudinal irradiated magnetoresistance turns out nearly the same irrespective of the sense of circular radiation.

## Introduction

Microwave-induced resistance oscillations and zero resistance states^1,2^ could be revealing a subtle novel form of coupling between radiation and matter. These effects show up in the longitudinal magnetoresistance (*R*_*xx*_) of a high mobility two-dimensional electron system (2DES) when irradiated under a perpendicular magnetic field (*B*) at low temperatures (*T*~1 *K*). Then, unexpected magnetoresistance oscillations rise up superimposed to the well-known longitudinal resistance profile. According to experiments^1,2^ the mobility of the sample has to be above 10^6^ *cm*^2^/*Vs* to get to observe them. To date, these oscillations have been obtained with microwave (MW) and terahertz (TH) radiation. When the radiation power (*P*) is increased, the amplitude of oscillations peaks and valleys increases as well and in the case of valleys, they turn into zero resistance states.

The available experimental evidence proves that these oscillations present some features that can be considered universal: they are periodic in *B*^−1^, present a 1/4 cycle phase shift in the oscillations minima^3^, are sensitive to temperature (T)^4,5^, present a non-linear increase with the radiation power (*P*) (squared root dependence)^6–16^, are immune to the sense of circular polarization in the incident radiation^17^, and other intriguing phenomena. Different theoretical models have been presented to try to explain the origin of these effects and their features^18–29^. Yet, after more than a decade of their discovery, they are still under debate and there is no clear consensus about their physical origin.

One of the most intriguing properties of irradiated magnetoresistance oscillations is the role of radiation polarization. According to the available experimental evidence, in the case of linear polarization there is some controversy about the dependence on polarization angle^4,7,17^. Nevertheless, in the case of circular polarization the experimental consensus is clear: the radiation-induced *R*_*xx*_ oscillations present immunity against the sense of circularly polarized radiation^17,30^. This is irrespective of radiation power^30^ and radiation frequency; this immunity has been detected also when using terahertz frequencies^31^. About the theoretical contributions on polarization immunity, some models conclude that the amplitude of the *R*_*xx*_ oscillations changes with the kind of polarization^20^ (left-handed or right-handed) showing no immunity. On the other hand there are other theoretical models that offer a physical explanation to this immunity^32,33^.

In this article we present a microscopic theoretical approach on the effect of the sense of circularly polarized radiation on irradiated *R*_*xx*_ based on a previous theory, developed by the authors: *the radiation-driven electron orbit*^21–25^. This theory is partially based on the displacement model^18^ and shares with it that the interplay between charged impurity scattering and radiation is at the heart of the radiation-induced *R*_*xx*_ oscillations. However it is different in the way that the radiation-matter interaction is worked out. According to this theory, the irradiated Landau state (LS) is spatially driven by radiation following a classical trajectory given by the solution of the driven classical oscillator. Then, the interaction of the driven-LS with charged impurities ends up giving rise to shorter and longer average advanced distances by the scattered electrons. These shorter and longer distances are reflected on irradiated *R*_*xx*_ as valleys and peaks respectively. As a first result of the theory, in an infinite sample the guiding center classical path would be different according to the the sense of circular polarization and thus, we would obtain different results in irradiated *R*_*xx*_. However, the limited size of the 2D sample with the key influence of the edges and the concurrent presence of DC electric fields (driving electric field and the Hall electric field) tend to quench or normalize the displacement of the driven guiding center making nearly equal both trajectories (left and right). In the end and in the presence of charged impurity scattering the radiation-induced oscillations turn out nearly the same irrespective of the sense of circular polarization.

## Theoretical Model

We consider a high mobility 2DES in the *x*−*y* plane subjected to a static and perpendicular magnetic field, and a DC electric field (driving electric field) parallel to the *x* direction, *E*_*dc*_. This system is irradiated with circularly polarized radiation, thus, we initially consider for left-handed circular polarization, (*E*_*x*_ = *E*_*y*_), the radiation electric field:

1 $$\overrightarrow{E}(t)={E}_{x}\,\overrightarrow{x}\,\sin \,wt-{E}_{y}\,\overrightarrow{y}\,\cos \,wt$$

where *E*_*x*_ and *E*_*y*_ are the electric field amplitudes of the corresponding components of $\overrightarrow{E}(t)$ and $\overrightarrow{x}$ and $\overrightarrow{y}$ are unitary vectors in the *x* and *y* directions. *w* is the frequency of the radiation field. Then, the total hamiltonian *H*, considering the symmetric gauge for the vector potential of *B*: $(\overrightarrow{{A}_{B}}=-\,\frac{1}{2}\overrightarrow{r}\times \overrightarrow{B})$, reads:

2 $$\begin{array}{lll}H & = & \frac{{P}_{x}^{2}+{P}_{y}^{2}}{2{m}^{\ast }}+\frac{{w}_{c}}{2}{L}_{z}+\frac{1}{2}{m}^{\ast }{[\frac{{w}_{c}}{2}]}^{2}[{(x-X\mathrm{(0))}}^{2}+{y}^{2}]\\  &  & -\frac{{e}^{2}{E}_{dc}^{2}}{2{m}^{\ast }{[\frac{{w}_{c}}{2}]}^{2}}-[x-X\mathrm{(0)]}\,e{E}_{x}\,\cos \,wt-ye{E}_{y}\,\sin \,wt\\  &  & -X\mathrm{(0)}e{E}_{0}\,\cos \,wt\end{array}$$

*X*(0) is the x-coordinate of the LS guiding center: $X\mathrm{(0)}=\frac{e{E}_{dc}}{{m}^{\ast }{({w}_{c}\mathrm{/2)}}^{2}}$, *e* is the electron charge, *w*_*c*_ is the cyclotron frequency and *L*_*z*_ is z-component of the electron total angular momentum. The important terms in this hamiltonian are^34,35^: the first is a kinetic term in the x-y plane, the second term is just the energy of the magnetic moment due to the orbital motion in the magnetic field, the third term corresponds to a 2D harmonic oscillator, and finally the term [−*xeE*_*x*_*coswt* − *yeE*_*y*_*sinwt*] represents the electromagnetic field. After some algebra^24^, the total wave function can be analytically obtained:

3 $${\rm{\Psi }}(x,y,t)\propto {\varphi }_{N}[(x-X\mathrm{(0)}-{x}_{c}(t)),(y-{y}_{c}(t)),t]$$

where *φ*_*N*_ are analytical solutions for the Schrödinger equation of a two-dimensional harmonic oscillator (electron under static magnetic field with the symmetric gauge). In polar coordinates the expression for *φ*_*N*_(*r*, *θ*, *t*)^35^:

4 $${\varphi }_{N}=\sqrt{\frac{n!}{2\pi {l}_{B}^{2}{2}^{|m|}{l}_{B}^{\mathrm{2|}m|}(n+|m|)!}}{r}^{|m|}{e}^{-im\theta }{L}_{n}^{|m|}(\frac{{r}^{2}}{2{l}_{B}^{2}}){e}^{-(\frac{{r}^{2}}{4{l}_{B}^{2}})}$$

where *n* is the radial quantum number, *m* is the angular momentum quantum number, ${L}_{n}^{|m|}$ are the associated Laguerre polynomials and ${l}_{B}=\sqrt{\frac{\hslash }{eB}}$ is the effective magnetic length. For the polar coordinates:

5 $$r{e}^{i\theta }=[x-X\mathrm{(0)}-{x}_{c}(t)]+i[y-{y}_{c}(t)]$$

*x*_*c*_(*t*) and *y*_*c*_(*t*) are the new coordinates of the guiding center of the radiation-driven LS and are obtained from a system of two coupled classical equations that turn up when solving the previous time dependent Schrodinger equation:

6 $$\frac{d{v}_{x}}{dt}+{w}_{c}{v}_{y}=\frac{e{E}_{x}}{{m}^{\ast }}\,\sin \,wt$$

7 $$\frac{d{v}_{y}}{dt}-{w}_{c}{v}_{x}=-\,\frac{e{E}_{y}}{{m}^{\ast }}\,\cos \,wt$$

where *v*_*x*_ and *v*_*y*_ are the components of the guiding center velocity when driven by radiation. Thus, the coordinates of the guiding center are calculated by integrating, ${v}_{x}=\frac{d{x}_{c}(t)}{dt}$ and ${v}_{y}=\frac{d{y}_{c}(t)}{dt}$. Then, finally the expressions read,

8 $${x}_{c}(t)=\frac{e{E}_{x}\,\sin \,wt}{{m}^{\ast }\sqrt{{w}^{2}{({w}_{c}-w)}^{2}+{\gamma }^{4}}}+[\frac{{w}_{c}}{w}]\frac{e{E}_{y}\,\sin \,wt}{{m}^{\ast }\sqrt{{w}^{2}{({w}_{c}-w)}^{2}+{\gamma }^{4}}}$$

9 $${y}_{c}(t)=[\frac{{w}_{c}}{w}]\frac{e{E}_{x}\mathrm{[1}-\,\cos \,wt]}{{m}^{\ast }\sqrt{{w}^{2}{({w}_{c}-w)}^{2}+{\gamma }^{4}}}+\frac{e{E}_{y}\mathrm{[1}-\,\cos \,wt]}{{m}^{\ast }\sqrt{{w}^{2}{({w}_{c}-w)}^{2}+{\gamma }^{4}}}$$

where *γ* is a damping factor for the electronic interaction with the lattice ions giving rise to acoustic phonons.

The obtained expressions for *x*_*c*_(*t*) and *y*_*c*_(*t*) correspond, according to our model, to left-handed circularly polarized radiation or cyclotron-resonance active condition (CRA). In our case the right-handed circularly polarized radiation corresponds to the cyclotron-resonance inactive condition (CRI). In the present theoretical model, the left-handed circularly polarized radiation corresponds to the cyclotron-resonance active condition or CRA at positive magnetic field because the radiation polarization direction is the same as the cyclic motion of electrons under the positive magnetic field. In the same way, the cyclotron-resonance inactive condition or CRI corresponds to the right-handed circular radiation because at positive magnetic field the radiation polarization direction is against the cyclic motion of electrons.

In the CRI case the radiation electric field is given by

10 $$\overrightarrow{E}(t)={E}_{x}\overrightarrow{x}\,\sin \,wt+{E}_{y}\overrightarrow{y}\,\cos \,wt$$

and thus, the system of two coupled equations that are obtained from the Schrodinger equation are given by,

11 $$\frac{d{v}_{x}}{dt}+{w}_{c}{v}_{y}=\frac{e{E}_{x}}{{m}^{\ast }}\,\sin \,wt$$

12 $$\frac{d{v}_{y}}{dt}-{w}_{c}{v}_{x}=\frac{e{E}_{y}}{{m}^{\ast }}\,\cos \,wt$$

And when solving this system we obtain for the guiding center coordinates for the CRI condition:

13 $${x}_{c}(t)=\frac{e{E}_{x}\,\sin \,wt}{{m}^{\ast }\sqrt{{w}^{2}{({w}_{c}-w)}^{2}+{\gamma }^{4}}}-[\frac{{w}_{c}}{w}]\frac{e{E}_{y}\,\sin \,wt}{{m}^{\ast }\sqrt{{w}^{2}{({w}_{c}-w)}^{2}+{\gamma }^{4}}}$$

14 $${y}_{c}(t)=[\frac{{w}_{c}}{w}]\frac{e{E}_{x}\mathrm{[1}-\,\cos \,wt]}{{m}^{\ast }\sqrt{{w}^{2}{({w}_{c}-w)}^{2}+{\gamma }^{4}}}-\frac{e{E}_{y}\mathrm{[1}-\,\cos \,wt]}{{m}^{\ast }\sqrt{{w}^{2}{({w}_{c}-w)}^{2}+{\gamma }^{4}}}$$

According to the above expressions of *x*_*c*_(*t*) and *y*_*c*_(*t*), for both CRA and CRI conditions, the guiding center of LS performs a classical circular trajectory driven by radiation. Both coordinates, *x*_*c*_(*t*) and *y*_*c*_(*t*), are made up of two contributions, the first one comes from the coupling with radiation along the x-axis and the second one comes from the coupling along the y-axis. This interesting result is the solution of the system of two coupled classical equations where the two coordinates of the guiding center velocity, *v*_*x*_(*t*) and *v*_*y*_(*t*) appear simultaneously in the two equations of the system. Then, this coupling implies that anything affecting the dynamics of the x-direction (for instance *v*_*x*_(*t*) or *v*_*y*_(*t*)) will affect the dynamics of the y-direction and viceversa.

Now, writing in one equation the two expressions for the two possible modes, active CRA and inactive CRI, for *x*_*c*_(*t*), (the one of interest to calculate longitudinal conductivity (*σ*_*xx*_) and *R*_*xx*_ we can get to:

15 $${x}_{c}(t)=\frac{e{E}_{x}\,\sin \,wt}{{m}^{\ast }\sqrt{{w}^{2}{({w}_{c}-w)}^{2}+{\gamma }^{4}}}\pm [\frac{{w}_{c}}{w}]\frac{e{E}_{y}\,\sin \,wt}{{m}^{\ast }\sqrt{{w}^{2}{({w}_{c}-w)}^{2}+{\gamma }^{4}}}$$

where the + sing corresponds to the CRA mode and the − to the CRI mode. Thus, the second term makes the difference between the two senses of circular radiation. The bigger this term, the bigger will be difference in irradiated *R*_*xx*_ between CRA and CRI; the experimentally observed circularly polarized radiation immunity of irradiated *R*_*xx*_ will depend, according to our theory, on this term.

**Figure 1:**
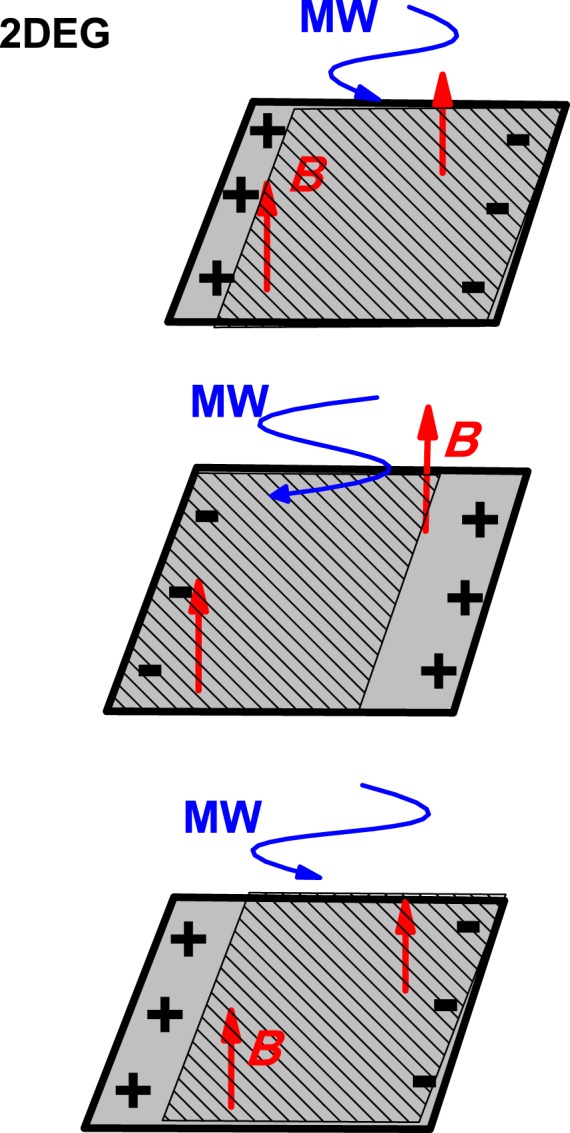
Schematic diagrams showing the dynamics of a two-dimensional electron system driven by radiation. The figures shown here represent only the spatial effect of radiation on the 2DES. The latter is being spatially driven when illuminated by microwave radiation. The radiation-driven oscillating 2D electron gas together with the existence of sample edges produce an alternating change in the position of the charged stripes at every end of the sample: an alternate electric field inside the 2D sample that goes against the radiation-driven trajectory of the LS guiding center. This effect slows down the motion of the driven-2DES.

The result of Eq. 15 corresponds to a 2D infinite sample, but in a real case the limited size of the sample, with the existence of edges in x and y directions, and the concurrence of the driving DC-electric field (responsible of the current in the x-direction) and the Hall electric field, will necessary alter the displacement of the guiding center. Besides, another important point that stems from the radiation-driven electron orbits model, can affect also the dynamics of the driven-LS guiding center in conjunction with the existence of sample edges: the spatial shift of the whole 2DES driven by radiation with respect to the fixed positive background of the lattice ions. This effect gives rise to the appearance of areas of opposite and alternate charge on either end of the sample creating electrostatic repulsion and attraction interactions (see Fig. 1). In other words, an alternate electric field inside the 2D sample that tends to go against the radiation-driven displacement of the LS guiding center. All of the above sample edge-related effects make the guiding center to slow down in its classical trajectory. The general outcome is a built-in quenching or damping effect on the radiation-driven motion of the guiding center making nearly equal or very similar the coordinate *x*_*c*_(*t*) of both CRA and CRI modes. To reflect this we propose at this point a phenomenological model where we introduce two damping factors affecting the amplitudes *E*_*x*_ and *E*_*y*_ and the corresponding delays in the sine terms (similar to the case of classical damped oscillators). Now and in the most general approach, we consider that this damping can be of different intensity for the x and y contribution to *x*_*c*_(*t*). For instance, we can physically justify this asymmetry in the damping, by the higher intensity of the Hall field compared to the DC-driving field; the point is that the motion along the y-direction, parallel to the Hall field, could be more hampered by the presence of this field than the one in the x-direction.

Thus, the total expression of *x*_*c*_(*t*) would be given by

16 $${x}_{c}(t)=\frac{{e}^{-{\gamma }_{x}t}e{E}_{x}\,\sin (wt+{\phi }_{x})}{{m}^{\ast }\sqrt{{w}^{2}{({w}_{c}-w)}^{2}+{\gamma }^{4}}}\pm [\frac{{w}_{c}}{w}]\frac{{e}^{-{\gamma }_{y}t}e{E}_{y}\,\sin (wt+{\phi }_{y})}{{m}^{\ast }\sqrt{{w}^{2}{({w}_{c}-w)}^{2}+{\gamma }^{4}}}$$

where *γ*_*x*_, *γ*_*y*_ and damping terms and *φ*_*x*_ and *φ*_*y*_ are phase differences. All of them are phenomenologically introduced according to our model. We can simplify the latter equation summing up the two terms considering that for left-handed circular radiation, *E*_*x*_ = *E*_*y*_ = *E*_0_:

17 $${x}_{c}(t)=[\frac{\sqrt{{e}^{-2{\gamma }_{x}t}{w}^{2}+{e}^{-2{\gamma }_{y}t}{w}_{c}^{2}\pm 2w{w}_{c}{e}^{-({\gamma }_{x}+{\gamma }_{y})t}\,\cos \,\phi }}{w}]\frac{e{E}_{0}\,\sin (wt+\alpha )}{{m}^{\ast }\sqrt{{w}^{2}{({w}_{c}-w)}^{2}+{\gamma }^{4}}}$$

If now and according to our phenomenological model, ${e}^{-{\gamma }_{y}t}=a{e}^{-{\gamma }_{x}t}$, where *a* < 1 assuming that the sample edge damping is more intense in the y-direction than in the x-direction, then we can finally write for *x*_*c*_(*t*),

18 $${x}_{c}(t)=[\frac{\sqrt{{w}^{2}+{a}^{2}{w}_{c}^{2}\pm 2aw{w}_{c}\,\cos \,\phi }}{w}]\frac{e{E}_{0}{e}^{-{\gamma }_{x}t}\,\sin (wt+\alpha )}{{m}^{\ast }\sqrt{{w}^{2}{({w}_{c}-w)}^{2}+{\gamma }^{4}}}$$

19 $$=\,{A}^{\ast }{e}^{-{\gamma }_{x}t}\,\sin (wt+\alpha )$$

where the amplitude A* is given by:

20 $${A}^{\ast }=[\frac{\sqrt{{w}^{2}+{a}^{2}{w}_{c}^{2}\pm 2aw{w}_{c}cos\phi }}{w}]\frac{e{E}_{0}}{{m}^{\ast }\sqrt{{w}^{2}{({w}_{c}-w)}^{2}+{\gamma }^{4}}}$$

where *φ* = *φ*_*y*_ − *φ*_*x*_. The phase difference *α* can be left out with the appropriate time shift (all driven-LS are oscillating in phase). And the total electronic orbit center coordinate in the x-direction, *X*(*t*), changes according to our model by^29^

21 $$\begin{array}{rcl}X(t) & = & X\mathrm{(0)}+{x}_{c}(t)\\  & = & X\mathrm{(0)}+{A}^{\ast }{e}^{-{\gamma }_{x}t}\,\sin \,wt\end{array}$$

Following the radiation-driven electron orbit model we can obtain the advanced distance by the electron due to charged impurity scattering when jumping between irradiated LS^29^:

22 $$\begin{array}{rcl}{\rm{\Delta }}X & = & {\rm{\Delta }}X\mathrm{(0)}-{A}^{\ast }{e}^{-{\gamma }_{x}\tau }\,\sin (w\tau )\\  & = & {\rm{\Delta }}X\mathrm{(0)}-{A}^{\ast }{e}^{-{\gamma }_{x}\frac{2\pi }{{w}_{c}}}\,\sin (2\pi \frac{w}{{w}_{c}})\end{array}$$

where Δ*X*(0) is the shift of the guiding center coordinate for the eigenstates involved in the scattering event when there is no light. According to the radiation-driven electron orbit model, the time $\tau =\frac{2\pi }{{w}_{c}}$ is the *flight time* or the time it takes the electron to jump from a Landau state to another one due to scattering.

Applying these last results to a Boltzmann transport model^34,36–38^, where *σ*_*xx*_ is given by:

23 $${\sigma }_{xx}={e}^{2}{\int }_{0}^{\infty }\,dE{\rho }_{i}(E)[{\rm{\Delta }}X{]}^{2}{W}_{I}(-\,\frac{df(E)}{dE})$$

being *E* the energy, *ρ*_*i*_(*E*) the density of initial states, *f*(*E*) the electron distribution function and *W*_*I*_ the charged impurity scattering rate, we can get to a final expression for *σ*_*xx*_^29,34,36–38^,

24 $${\sigma }_{xx}\propto {[{\rm{\Delta }}{X}^{0}-{A}^{\ast }{e}^{-{\gamma }_{x}\frac{2\pi }{{w}_{c}}}\sin (2\pi \frac{w}{{w}_{c}})]}^{2}[1+{e}^{\frac{-\pi {\rm{\Gamma }}}{\hslash {w}_{c}}}\frac{{X}_{S}}{\sinh \,{X}_{S}}\,\cos (2\pi \frac{{E}_{F}}{\hslash {w}_{c}})]$$

where Γ is the LS width, *E*_*F*_ stands for the Fermi energy and ${X}_{S}=\frac{2{\pi }^{2}{k}_{B}T}{\hslash {w}_{c}}$, *k*_*B*_ being the Boltzmann constant. To obtain *R*_*xx*_ we use the relation ${R}_{xx}=\frac{{\sigma }_{xx}}{{\sigma }_{xx}^{2}+{\sigma }_{xy}^{2}}\simeq \frac{{\sigma }_{xx}}{{\sigma }_{xy}^{2}}$, where ${\sigma }_{xy}\simeq \frac{{n}_{i}e}{B}$ and ${\sigma }_{xx}\ll {\sigma }_{xy}$, *n*_*i*_ being the 2D electron density.

Within a more general approach we could extend to the Hall resistance, *R*_*xy*_, what we have applied to *R*_*xx*_. According to the model the charge strips would be changing with radiation in x and y direction and this should affect the Hall voltage and the Hall resistance. Thus, a possible effect on the Hall resistance would be an alternate voltage to be added to the total Hall voltage. The value of the Hall electric field is high and maybe the radiation effect on it could be very small. This would depend mainly of the radiation power.

## Results and Discussion

**Figure 2:**
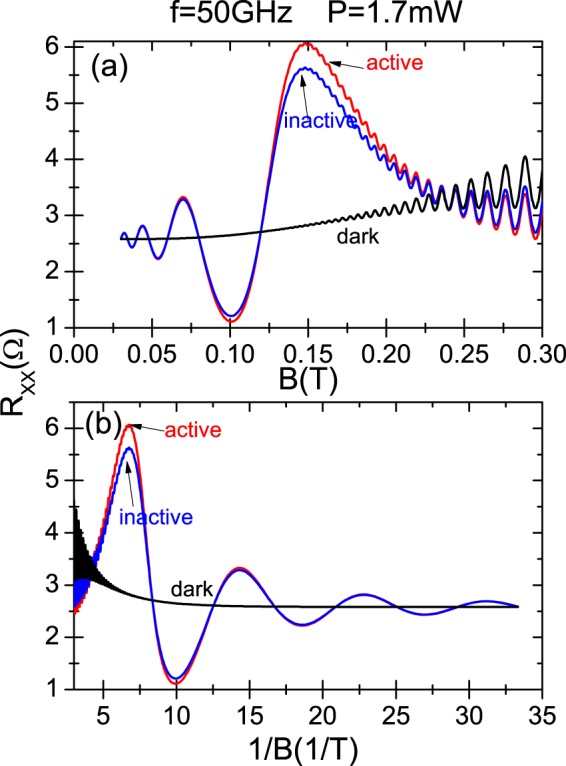
Calculated linear magnetoresistance vs magnetic field (upper panel) and vs the inverse of the magnetic field (lower panel) under circularly polarized radiation for CRA and CRI conditions. The radiation frequency is *f* = 50 GHz and temperature *T* = 1 K. The edge-damping *γ*_*y*_ = 0.2 × 10^10^ *s*^−1^ and the phase difference, *φ* = *π*/3. The dark case is displayed. For the irradiated curves, we observe that the radiation-induced oscillations are periodic in *B*^−1^ and the minima 1/4-cycle shifted. It is clearly observed that the irradiated magnetoresistivity response for the CRA and CRI conditions is nearly the same over the whole range of magnetic field.

**Figure 3:**
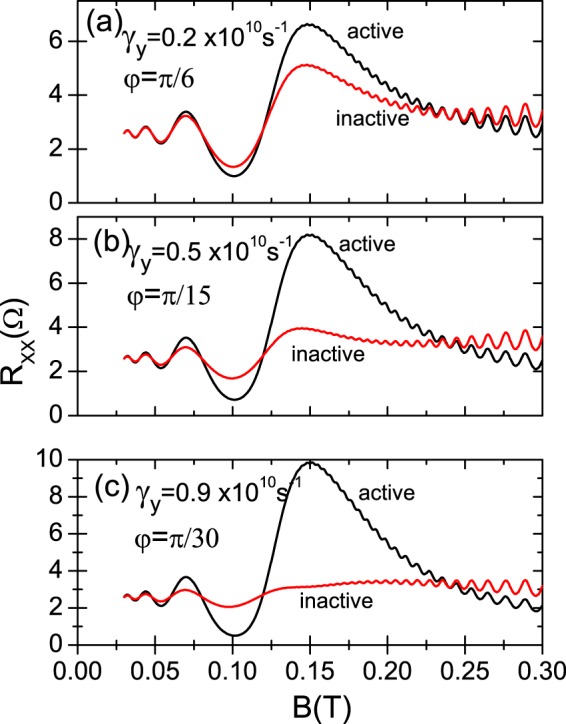
Calculated magnetoresistance under radiation vs magnetic field in three different panels for increasing values of the edge-damping *γ*_*y*_ and decreasing of the phase difference *φ*. In panel (a) *γ*_*y*_ = 0.2 × 10^10^ *s*^−1^ and *φ* = *π*/6, in panel (b) *γ*_*y*_ = 0.5 × 10^10^ *s*^−1^ and *φ* = *π*/15, and in panel (c) *γ*_*y*_ = 0.9 × 10^10^ *s*^−1^ and *φ* = *π*/30. We observe that as *γ*_*y*_ gets bigger and closer to *γ*_*x*_, and the phase difference *φ* gets smaller, the traces of both CRA and CRI increasingly diverge. Remarkably, the divergence of both conditions gets bigger as the magnetic field increases for each panel irrespective of the value of *γ*_*y*_ and *φ*. T = 1 K.

All the calculated curves presented in this section are based on Eq. 24 and on the tensor relation between *R*_*xx*_ and *σ*_*xx*_ (see above). In Fig. 2 we exhibit radiation-induced *R*_*xx*_ oscillations vs magnetic field under circularly polarized radiation for CRA and CRI modes. The radiation frequency is *f* = 50 GHz and temperature *T* = 1 K. The dark case is also presented. For the irradiated traces, we observe that the radiation-induced oscillations are periodic in *B*^−1^ and the minima are 1/4-cycle shifted. Interestingly enough, we observe that the irradiated magnetoresistivity response for the CRA and CRI conditions is nearly the same over the whole range of magnetic field. There seems to be a small difference around cyclotron resonance. The general calculated result is in qualitatively agreement with experiment^17,30^. In our simulations we have used experimental values for radiation power and frequency, temperature, electron density, type of sample, etc.^30^. Thus, using these experimental values and in order to achieve circular polarization immunity, we have obtained phenomenological values for the edge-damping ${\gamma }_{x}\simeq {10}^{10}\,{s}^{-1}$, ${\gamma }_{y}\simeq 0.1\times {10}^{10}\,{s}^{-1}$, and for the phase difference $\phi \simeq \pi \mathrm{/3}$. We can consider these values as the threshold between immunity and non-immunity scenarios according to experimental parameters^17,30^. In platforms with a lesser influence of the sample edges, we would obtain a clear difference between irradiated *R*_*xx*_ for CRA and CRI modes. This is what we display in Fig. 3, where it is exhibited calculated magnetoresistance under radiation vs magnetic field in three different panels for three different values of the edge-damping *γ*_*y*_ and *φ* and same value of *γ*_*x*_. In panel (a) *γ*_*y*_ = 0.2 × 10^10^ *s*^−1^ and *φ* = *π*/6, in panel (b) *γ*_*y*_ = 0.5 × 10^10^ *s*^−1^ and *φ* = *π*/15, and in panel (c) *γ*_*y*_ = 0.9 × 10^10^ *s*^−1^ and *φ* = *π*/30. It is clear that as *γ*_*y*_ gets bigger and closer to *γ*_*x*_, and the phase difference *φ* gets smaller, the traces of both CRA and CRI increasingly diverge: the CRA trace increases and the CRI decreases. And in the same way, this divergence gets bigger as the magnetic field increases. This latter behavior is qualitatively similar for the three panels irrespective of the value of *γ*_*y*_ and *φ*.

**Figure 4:**
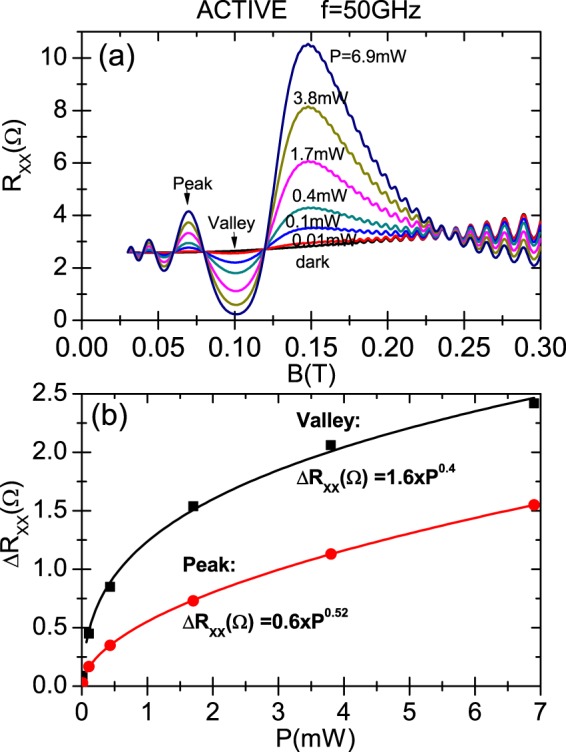
Dependence on radiation power *P* of the calculated magnetoresistivity under circularly polarized light for the CRA condition. The edge-damping *γ*_*y*_ = 0.2 × 10^10^ *s*^−1^ and the phase difference, *φ* = *π*/3. In panel (a) irradiated *R*_*xx*_ as a function of *B*, for different radiation intensities starting from dark and for a radiation frequency of *f* = 50 GHz. We observed that the *R*_*xx*_ oscillations amplitudes increase with an increasing power. In panel (b) Δ*R*_*xx*_ = *R*_*xx*_(*light*) − *R*_*xx*_(*dark*) versus *P* for *B* corresponding to the peak and valley labels of panel (a). For both sets of *R*_*xx*_ values we obtain a square root dependence as shown in the corresponding fits where the exponent of *P* is around 0.5. T = 1 K.

In Fig. 4 we present the dependence of calculated irradiated *R*_*xx*_ on *P* for the CRA condition. In panel (a) we display *R*_*xx*_ vs magnetic field for a radiation frequency of 50 GHz whereas the power ranges from 0.01 mW to 6.9 mW. In between we also exhibit traces of 0.1, 0.4, 1.7 and 3.8 mW. The dark case is also presented. The temperature used is *T* = 1 K. The sample edge damping parameters correspond to the ones of Fig. 2, i.e., a scenario of circular polarization immunity. We observe, as expected, that magnetoresistance oscillations increase their amplitudes as *P* increases from dark. In panel (b) we exhibit, for the same radiation frequency Δ*R*_*xx*_ = *R*_*xx*_(*light*) − *R*_*xx*_(*dark*) versus *P* for the magnetic fields labelled in panel (a) with peak and valley. The power dependence of the oscillatory *R*_*xx*_ clearly indicates a non-linear behavior. We want to check out the previously obtained sublinear power law for the dependence of irradiated *R*_*xx*_ on *P*. In this way we obtain for both sets of *R*_*xx*_ values, according to the calculated fits, an approximately square root dependence on *P*. For the valley trace we obtain a fit given by Δ*R*_*xx*_ = 1.6 × *P*^0.4^ and for the peak Δ*R*_*xx*_ = 0.6 × *P*^0.52^; for both cases the exponent is around 0.5 that implies a square root dependence. We can theoretically explain these results according to the radiation-driven electron orbit model: in the expression of *σ*_*xx*_ and then in *R*_*xx*_, *P* only shows up in the numerator of the amplitude A* as $\sqrt{P}\propto {E}_{0}$, the radiation electric field. Thus, on the one hand, *P* does not affect the phase of *R*_*xx*_ oscillations that remains constant as *P* changes, and on the other hand ${R}_{xx}\propto \sqrt{P}$, giving rise to the sublinear (square root) power law for the dependence of *R*_*xx*_ on *P*.

**Figure 5:**
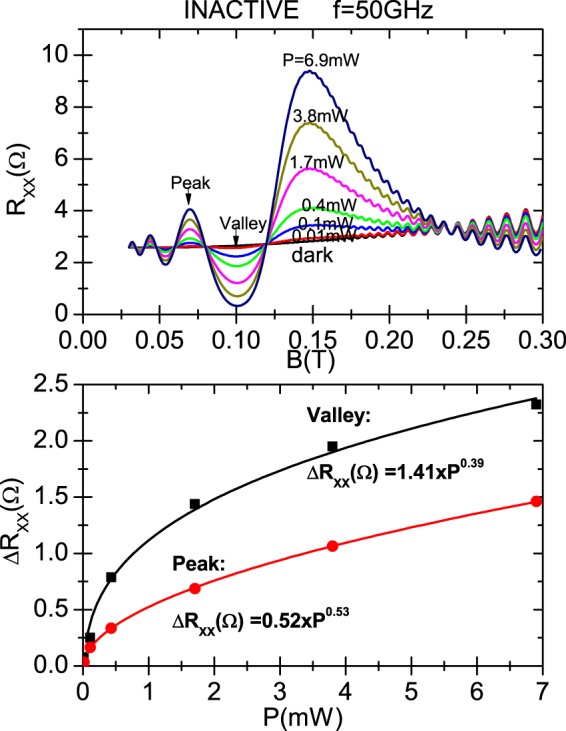
Same as in Fig. 4 but for the CRI condition. The edge-damping *γ*_*y*_ = 0.2 × 10^10^ *s*^−1^ and the phase difference, *φ* = *π*/6. In panel (a) traces of irradiated *R*_*xx*_ vs B for the same radiation powers as in Fig. 4. All of them are nearly the same as the ones of CRA condition proving circular polarization immunity. In panel (b) Δ*R*_*xx*_ = *R*_*xx*_(*light*) − *R*_*xx*_(*dark*) versus *P* for the magnetic fields labelled in panel (a) with peak and valley. We obtain for both sets of *R*_*xx*_ values calculated fits showing approximately square root dependence on *P*. T = 1 K.

In Fig. 5 we present the same as in Fig. 4 but for the CRI condition. In panel (a) we show traces of irradiated *R*_*xx*_ vs magnetic field for the same radiation powers as Fig. 4. All of them are nearly the same as the ones of CRA condition proving circular polarization immunity except for a region of magnetic fields around the cyclotron resonance. In panel (b) we exhibit Δ*R*_*xx*_ = *R*_*xx*_(*light*) − *R*_*xx*_(*dark*) versus *P* for the magnetic fields labelled in panel (a) with peak and valley. We obtain for both sets of *R*_*xx*_ values calculated fits that show approximately a square root dependence on *P* too. For the valley trace we obtain a fit given by Δ*R*_*xx*_ = 1.41 × *P*^0.39^ and for the peak Δ*R*_*xx*_ = 0.52 × *P*^0.53^. Thus, as in the active condition, we obtain exponents for *P* around 0.5. This proves a square root dependence as predicted by the radiation-driven electron orbit model.

Another remarkable result that is obtained from Figs 4 and 5 is that the immunity of radiation-induced magnetoresistance oscillations to the orientation of the circular polarization holds independently of radiation power. This an expected result according to Eqs 20 and 24 of the model. According to them, it is clear that the power variation would affect in the same way both circular polarization conditions, CRA and CRI.
